# Impact of invasive fungal infection on outcomes of severe sepsis: a multicenter matched cohort study in critically ill surgical patients

**DOI:** 10.1186/cc6766

**Published:** 2008-01-16

**Authors:** Guo-Hao Xie, Xiang-Ming Fang, Qiang Fang, Xin-Min Wu, Yu-Hong Jin, Jun-Lu Wang, Qu-Lian Guo, Miao-Ning Gu, Qiu-Ping Xu, Dong-Xin Wang, Shang-Long Yao, Shi-Ying Yuan, Zhao-Hui Du, Yun-Bo Sun, Hai-Hong Wang, Shui-Jing Wu, Bao-Li Cheng

**Affiliations:** 1 Department of Anesthesiology and Intensive Care Unit, the First Affiliated Hospital, School of Medicine, Zhejiang University, 79 Qingchun Road, 310003, Hangzhou, China; 2 Department of Anesthesiology and Intensive Care Unit, the First Affiliated Hospital, School of Medicine, Peking University, 8 West Shenku Avenue, 100034, Beijing, China; 3 Intensive Care Unit, Lihuili Hospital, School of Medicine, Ningbo University, 57 Xingning Road, 315040, Ningbo, China; 4 Department of Anesthesiology, the First Affiliated Hospital, Wenzhou Medical College, 2 Fuxue Road, 325000, Wenzhou, China; 5 Department of Anesthesiology and Intensive Care Unit, Xiangya Hospital, Xiangya Medical College, Central South University, 87 Xiangya Road, 410008, Changsha, China; 6 Department of Anesthesiology and Intensive Care Unit, South Hospital, South Medical University, 1836 Guangzhou Road, 510515, Guangzhou, China; 7 Department of Anesthesiology and Intensive Care Unit, Sir Run Run Shaw Hospital, School of Medicine, Zhejiang University, 3 Qingchun Road, 310016, Hangzhou, China; 8 Department of Anesthesiology and Intensive Care Unit, Union Hospital, Tongji Medicine College, Huazhong University of Science and Technology, 1095 Jiefang Road, 430030, Wuhan, China; 9 Intensive Care Unit, Zhongnan Hospital, School of Medicine, Wuhan University, 169 Donghu Road, 430071, Wuhan, China; 10 Department of Anesthesiology and Intensive Care Unit, Qingdao University Hospital, School of Medicine, Qingdao University, 16 Jiangsu Road, 266011, Qingdao, China

## Abstract

**Introduction:**

Fungal infection is increasingly common in critical illness with severe sepsis, but the influence of invasive fungal infection (IFI) on severe sepsis is not well understood. The aim of this study was to investigate the impact that IFI has on the outcomes of critically ill surgical patients with severe sepsis in China by means of matched cohort analysis; we also evaluated the epidemiologic characteristics of IFI in this population.

**Methods:**

Records for all admissions to 10 university hospital surgical intensive care units (ICUs) from December 2004 to November 2005 were reviewed. Patients who met criteria for severe sepsis were included. IFI was identified using established criteria based on microbiologic or histological evidence. A matched cohort study was conducted to analyze the relationship between IFI and outcomes of severe sepsis.

**Results:**

A total of 318 patients with severe sepsis were enrolled during the study period, of whom 90 (28.3%) were identified as having IFI. A total of 100 strains of fungi (58% *Candida albicans*) were isolated from these patients. Independent risk factors for IFI in patients with severe sepsis included mechanical ventilation (>3 days), Acute Physiology and Chronic Health Evaluation score, coexisting infection with both Gram-positive and Gram-negative bacteria, and urethral catheterization (>3 days). Compared with the control cohort, IFI was associated with increased hospital mortality (*P *< 0.001), high hospital costs (*P *= 0.038), and prolonged stay in the ICU (*P *< 0.001) and hospital (*P *= 0.020).

**Conclusion:**

IFI is frequent in patients with severe sepsis in surgical ICUs and is associated with excess risk for hospital mortality, longer ICU and hospital stays, and greater consumption of medical resources.

## Introduction

Invasive fungal infection (IFI) is a severe clinical complication in immunocompromised patients, such as neutropenic patients, recipients of bone marrow or solid organ transplants, cancer patients receiving chemotherapy, and HIV-infected patients. However, during the past two decades, with advances in diagnostic and therapeutic interventions, critically ill patients with lesser degrees of immunocompromise, especially those in surgical and neonatal intensive care units (ICUs), have emerged as another population at high risk for IFI [1-3].

Sepsis is the body's systemic inflammatory response to infection. It is considered severe when it is associated with acute organ dysfunction. According to epidemiologic studies [4-13], severe sepsis has become a leading cause of morbidity and mortality in critical illness, and etiologic evidence [14] indicates that the incidence of fungal infection in septic patients is increasing. Fungal organisms are common pathogens in surgical patients and patients suffering from severe sepsis. Raymond and coworkers [15] observed that fungal infection accounted for 12.3% of all episodes of surgical infection in a study conducted over 38 months. Finfer and colleagues [8] reported that fungi accounted for approximately 12.1% of all microbial isolates in their study of severe sepsis among adult admissions to ICUs in Australia and New Zealand [8]. Recently, the large pan-European Sepsis Occurrence in Acutely Ill Patients (SOAP) study [4] reported that fungal infection was observed in 17% of all septic patients in European ICUs. Furthermore, IFI was found to be associated with excess mortality, hospital stay, and cost [16,17]. Annane and coworkers [18] identified fungal infection as an independent risk factor for increased mortality among patients with septic shock. However, fungal infection was not found to be among the significant predictors of fetal outcome in some other studies [4].

In the present study we attempted to determine the impact that IFI has on outcomes of severe septic patients in multiple surgical ICUs in China. We undertook a matched cohort study in which severe septic patients with and without IFI were matched for unit, age, sex and severity of illness.

## Materials and methods

### Study population and data collection

The present study was conducted in the surgical ICUs of 10 university hospitals in six major provinces from 1 December 2004 to 30 November 2005 (participating centers are listed under Acknowledgement [see below]). The study protocol was approved by the ethical committee in each participating center, and informed consent was waived because of the observational nature of this study. All adult patients (age ≥18 years) admitted to the participating surgical ICUs during the period of observation and who met criteria for severe sepsis were included in the study cohort. These patients were evaluated prospectively by investigators daily by chart review and interview of ICU physicians. For all enrolled patients, the following data were collected: age, sex, primary diagnosis, chronic comorbidities, clinical data needed for calculation of Acute Physiology and Chronic Health Evaluation (APACHE) II score during the first 24 hours after ICU admission [19], daily Sequential Organ Failure Assessment (SOFA) [20,21], microbiological and clinical infection data, antibiotics and antifungal agents administered, hospital costs, and hospital outcome. The chronic comorbidity system in this study was constructed by selecting ICD-9-CM codes suggestive of chronic disease within separate organ systems. 'Cost' was defined as the total expenditures on medical care attributable to a patient in the hospital, including charges for medical care, nursing, medication, and laboratory testing. Indirect expenses were not calculated.

Those who were readmitted and had been included on their first admission were not included for a second time. If one patient suffered more than one episode of IFI, then only the first episode was included in the study.

For the matched cohort study, patients with severe sepsis suffering IFI during their ICU stay were matched to control patients with severe sepsis but without IFI. We attempted to match each individual with severe sepsis with a control patient in the same center. The matching process included the following factors: sex, age (± 10 years) and APACHE II score (± 3) [22]. If one patient could be matched to two or more control patients, then the control patient with the closest APACHE II score was selected.

### Definitions

The criteria for IFI were defined based on the study conducted by and coworkers [23]. This definition requires the presence of fungemia, specifically blood culture yielding fungi in patients with temporally related clinical signs and symptoms compatible with relevant organism. It also requires IFI to be present in other sites, to be confirmed histopathologically or cytopathologically in needle aspirate or biopsy specimen, or fulfilling the following four criteria: positive culture result for samples obtained via sterile procedure from normally sterile sites, excluding urine and mucous membranes; compatible clinical and radiologic manifestations; no evidence of infection caused by micro-organisms other than fungus; and improvement in signs and symptoms, and radiology after use of antifungal agents. Treated as a special case, invasive pulmonary aspergillosis was diagnosed only if there was positive histologic testing and/or culture after biopsy or autopsy, as defined by Vandewoude and coworkers [24]. Detection of fungi and speciation of isolates were performed at the laboratories of participating hospitals, in accordance with their standard protocols. The antifungal activity of amphotericin B, fluconazole and itraconazle was also tested in these laboratories. The time of onset of IFI was defined as the date in which the first positive culture or biopsy was identified.

Sepsis was defined in accordance with the American College of Chest Physicians/Society of Critical Care Medicine consensus conference definition [25]. Severe sepsis was defined as sepsis plus sepsis-induced acute organ dysfunction (occurring in at least one organ), as indicated by a SOFA score of 2 or more for the organ in question.

'Combination antibiotic therapy' in this report refers to antibiotic therapy involving more than one of the following types of antibiotic, administered daily for at least 3 days: penicillins, cephalosporins, carbapenem, macrolides, glycopeptides, aminoglycosides, quinolones, sulfonamides, and others.

### Statistical analyses

Continuous data are presented as median with interquartile range (25th to 75th percentiles) and compared using Mann-Whitney test, except for the variable cost, which is presented as mean ± standard and compared using the *t *test. Categorical data are shown as percentages and compared using the χ^2 ^test. In the matched cohort study, comparisons of paired baseline characteristics were performed using the paired Student's *t*-test and the McNemar test for continuous and categorical variables, respectively. The matched risk ratio and excess risk were expressed by ratio and difference in mortality rates between exposed and unexposed patients, respectively. The tests were two-sided, and *P *< 0.05 wa deemed to reflect statistical significance.

A multivariate nonconditional logistic regression analysis was conducted in patients with severe sepsis, with occurrence of IFI in the ICU as a dependent factor. Variables considered in multivariate modeling included age, sex, chronic comorbidity, coexisting bacterial infection, APACHE II score, mechanical ventilation (>3 days), central venous catheterization (>3 days), urethral catheterization (>3 days), and combination antibiotic therapy in the ICU (for patients with severe sepsis and IFI, only the data before the onset of IFI were adopted in the regression). Forward elimination, which employed a combination of the procedures used in the forward entry and backward removal methods, was adopted in the regression analysis. The effect on occurrence of IFI in patients with severe sepsis was considered statistically significant if the regression coefficient associated with IFI was at a level of *P *< 0.05. Statistical analysis was conducted using SPSS 13.0 for Windows (SPSS Inc., Chicago, IL, USA).

## Results

### Incidence and demographics

From 1 December 2004 to 30 November 2005, 318 patients were identified as having severe sepsis and hence were enrolled in the study cohort, including 206 men (64.8%) and 112 women (35.2%). Among these 318 patients, 90 (28.3%) developed IFI in the ICU, including 20 (6.3%) suffering from fungemia. A comparison of demographic characteristics for the 90 patients with IFI versus those of patients without IFI (control patients) is presented in Table 1.

**Table 1:** Demographic characteristics of severe sepsis patients with and without invasive fungal infection

Variables	Severe sepsis patients with IFI (*n *= 90)	Severe sepsis patients without IFI (*n *= 228)	*P *value
Baseline descriptors			

Age (years)	65 (50–76.25)	61.5 (45–73)	NS
Sex (male; *n *[%])	57 (63.3%)	149 (65.4%)	NS
Admission APACHE II score (mean [IQR])	21 (17–27)	18 (13–23)	0.001
Admission SOFA score(mean [IQR])	8 (6–12)	8 (5–11.75)	NS
Comorbidity (*n *[%])	71 (78.9%)	165 (72.4%)	NS
Cancer (*n *[%])	14 (15.6%)	24 (10.5%)	NS
Diabetes mellitus (*n *[%])	15 (16.7%)	26 (11.4%)	NS
Interventions			
Mechanical ventilation (*n *[%])	71 (78.9%)	75 (32.9%)	<0.001
Central venous catheterization (*n *[%])	74 (82.2%)	122 (53.5%)	<0.001
Urinary catheterization (*n *[%])	84 (93.3%)	137 (60.1%)	<0.001
Arterial catheterization (*n *[%])	51 (56.7%)	112 (49.1%)	NS
Total parenteral nutrition (*n *[%])	49 (54.4%)	99 (43.4%)	NS
Corticosteroids or immunodepressant drugs (*n *[%])	22 (24.4%)	45 (19.7%)	NS
Renal replacement therapy (*n *[%])	21 (23.3%)	28 (12.3%)	0.024
Outcomes			
ICU LOS (days; mean [IQR])	16 (7.75–31)	5 (2–10)	<0.001
Hospital LOS (days; mean [IQR])	29.5 (17.75–50.25)	19 (10.25–36)	<0.001
Daily costs ($; mean ± SD)	520 ± 319	481 ± 437	NS
Hospital costs ($; mean ± SD)	17,051 ± 14,183	8,474 ± 9,484	0.001
Hospital mortality	61 (67.8%)	94 (41.2%)	<0.001

APACHE, Acute Physiology and Chronic Health Evaluation; ICU, intensive care unit; IQR, interquartile range; IFI, invasive fungal infection; LOS, length of stay; NS, not significant; SD, standard deviation; SOFA, Sequential Organ Failure Assessment.

### Characteristics of fungal infection

In all 90 patients with IFI, 100 episodes of invasive fungal infection were identified, including 58 involving *Candida albicans *(58.0%), 17 *C. tropicalis *(17.0%), 15 *C. glabrata *(15.0%), three *C. parapsilosis *(3.0%), three *Aspergillus *spp. (3.0%), and four other species or unclassified (4.0%). Eight patients were infected with two or more fungal species (Table 2). Resistance to fluconazole was detected in 24 isolates.

**Table 2:** Characteristics of fungal infection

Characteristics	Episodes (*n *[%])
Pathogens	

*Candida albicans*	58 (58.0)
*Candida tropicalis*	17 (17.0)
*Candida glabrata*	15 (15.0)
*Candida parapsilosis*	3 (3.0)
*Aspergillus*	3 (3.0)
Others or unclassified	4 (4.0)
Site of infection	
Lung	62 (56.4)
Abdomen	25 (22.7)
Bloodstream or catheter related	15 (13.6)
Other sites	8 (7.3)

Lung was the most common site of IFI, followed by abdominal, and bloodstream or catheter-related infection (Table 2). Twenty patients suffered from multi-site infection.

### Interventions

In all, 78.9%, 82.2%, and 93.3% of the 90 patients with IFI underwent mechanical ventilation, central venous catheterization, and urethral catheterization, respectively. However, patients with severe sepsis but without IFI were likely to undergo fewer invasive procedures tosse patients with IFI (Table 1).

Combination antibiotic therapies were adminstered to 45 (50.0%) of the 90 patients before the onset of IFI, which was not statistically significant from that in patients with severe sepsis but without IFI during the entire stay in the ICU (57.0%; *P *= 0.263). In the participating surgical ICUs, antifungal agents were chosen based on the results of antifungal susceptibility testing. Fluconazole was administered to 66 patients with IFI, whereas itraconazole was administered to 22 patients and amphotericin B to two patients. Also, 75 patients received intravenous antifungal agents.

### Predictors of invasive fungal infection

The variables retained in the final model in the multivariate logistic regression and hence associated with increased risk for IFI in patients with severe sepsis included the following: mechanical ventilation (>3 days), APACHE II score, coexisting infection with both Gram-positive and Gram-negative bacteria, and urethral catheterization (>3 days; Table 3).

**Table 3:** Logistic regression analysis in patients with severe sepsis, with occurrence of IFI in the ICU as the dependent factor

Variable	OR (95% CI)	*P *value
Mechanical ventilation (>3 days)	3.28 (1.67–6.45)	0.001
APACHE II score	1.43 (1.17–1.76)	0.001
Coexisting infection by both Gram-positive and Gram-negative bacteria	4.06 (2.23–7.38)	<0.001
Urethral catheterization (>3 days)	3.97 (1.45–10.89)	0.007

A total of 318 patients were included in this analysis. APACHE, Acute Physiology and Chronic Health Evaluation; APS, Acute Physiology Score; CI, confidence interval; OR, odds ratio.

### Mortality

The observed hospital mortality rate was significantly higher in patients with severe sepsis and IFI than in those patients without IFI (67.8% versus 41.2%; *P *< 0.001). There was no significant difference in hospital mortality between male and female patients with IFI (61.4% versus 51.5%; *P *= 0.384).

### Resource use and costs

The median hospital and ICU lengths of stay (LOSs) for all 318 patients with severe sepsis were 7 (3 to 14) days and 22 (12 to 39) days, respectively. Patients with severe sepsis and IFI had longer ICU and hospital LOSs than did those without IFI (ICU LOS: 16 [7.75 to 31] days versus 5 [2 to 10] days, *P *< 0.001; hospital LOS: 29.5 [17.75 to 50.25] days versus 19 [10.25 to 36.00] days, *P *< 0.001). Among patients with severe sepsis and IFI, the ICU and hospital LOSs for men were comparable to those for women (ICU LOS: 18 [8 to 30] days versus 13 [7 to 32] days, *P *= 0.505; hospital LOS: 28 [17 to 56] days versus 31 [20.5 to 47.5] days, *P *= 0.657).

The mean hospital cost was much higher in severe septic patients with IFI than those without IFI ($17,051 ± 14,183 versus $8,474 ± 9,484, *P *= 0.001). However, the mean daily costs were similar between the two groups ($520 ± 319 versus $481 ± 437, *P *= 0.59).

### Results of matched cohort study

Sixty patients with severe sepsis and IFI could be matched to 60 patients with severe sepsis but without IFI (matched control patients), based on unit, sex, age, and APACHE II score. The patients for whom matched control patients could be identified accounted for 66.7% of the 90 patients with severe sepsis and IFI, and these matched 60 patients with severe sepsis and IFI had similar mortality to that in the unmatched 30 patients (Table 4). Compared with control patients, markedly higher hospital mortality, ICU LOS, hospital LOS and hospital costs, and more aggressive interventions (mechanical ventilation, central venous catheterization, and urinary catheterization) were observed in matched patients with severe sepsis and IFI. However, daily costs were similar between the two groups (Table 5). IFI was associated with a matched excess risk for death in hospital of 20% (70.0% versus 50.0%, *P *= 0.023), and the matched risk ratio was 1.4.

**Table 4:** Comparison between matched and unmatched severe septic patients with IFI

Variables	Matched patients with IFI (*n *= 60)	Unmatched patients with IFI (*n *= 30)	*P *value
Age (years; mean [IQR])	67.5 (52.25–75.75)	62 (45.5–79.5)	NS
Sex (male; %)	71.7%	46.7%	0.019
Admission APACHE score (mean [IQR])	20 (16.25–25)	25 (17–32)	0.035
Admission SOFA score (mean [IQR])	8 (6–10.75)	9 (7–14)	NS
ICU LOS (days; mean [IQR])	17.5 (10–31)	13 (4.5–30.5)	NS
Hospital LOS (days; mean [IQR])	30 (19.25–50)	29 (16–55.5)	NS
Daily costs ($; mean ± SD)	526 ± 319	501 ± 332	NS
Hospital costs ($; mean ± SD)	16,804 ± 14,102	17,749 ± 15,081	NS
hospital mortality (%)	70.0%	63.3%	NS

APACHE, Acute Physiology and Chronic Health Evaluation; ICU, intensive care unit; IFI, invasive fungal infection; LOS, length of stay; NS, not significant; SD, standard deviation; SOFA, Sequential Organ Failure Assessment.

**Table 5:** Comparison between severe septic patients with and without IFI in the matched cohort study

Variable	Severe sepsis patients with IFI (*n *= 60)	Severe sepsis patients without IFI (*n *= 60)	*P *value
Baseline descriptors			

Age (years; mean [IQR])	67.5 (52.25–75.75)	68 (54–74)	NS
Admission APACHE II score (mean [IQR])	20 (16.25–25)	20 (16.25–23.75)	NS
Admission SOFA score (mean [IQR])	8 (6–10.75)	8 (5–12)	NS
Co-morbidity (*n *[%])	47 (78.3%)	48 (80.0%)	NS
Cancer (*n *[%])	9 (15.0%)	11 (18.3%)	NS
Diabetes mellitus (*n *[%])	10 (16.7%)	9 (15.0%)	NS
Interventions			
Mechanical ventilation (*n *[%])	49 (81.7%)	24 (40.0%)	<0.001
Central venous catheterization (*n *[%])	51 (85.0%)	35 (58.3%)	<0.001
Urinary catheterization (*n *[%])	58 (96.7%)	36 (60.0%)	<0.001
Arterial catheterization (*n *[%])	41 (68.3%)	35 (58.3%)	NS
Total parenteral nutrition (*n *[%])	35 (58.3%)	23 (38.3%)	0.036
Corticosteroids or immunodepressant drugs (*n *[%])	13 (21.7%)	16 (26.7%)	NS
Renal replacement therapy (*n *[%])	12 (20.0%)	11 (18.3%)	NS
Outcomes			
ICU LOS (days; mean [IQR])	17.5 (10–31)	6.5 (3–10)	<0.001
Hospital LOS (days; mean [IQR])	30 (19.25–50)	20 (10.25–38.5)	0.020
Mean daily costs ($; mean ± SD)	592 ± 326	469 ± 396	NS
Hospital costs ($; mean ± SD)	17,951 ± 15,470	10,023 ± 12,347	0.038
Hospital mortality (*n *[%])	42 (70.0%)	42 (50.0%)	0.023

APACHE, Acute Physiology and Chronic Health Evaluation; ICU, intensive care unit; IQR, interquartile range; IFI, invasive fungal infection; LOS, length of stay; NS, not significant; SD, standard deviation; SOFA, Sequential Organ Failure Assessment.

## Discussion

The present study focused on IFI in patients with severe sepsis in surgical ICUs, and it contributes important additional information to that from the large studies on epidemiology of severe sepsis published thus far. The study identified a 28.3% incidence of IFI in critically ill patients with severe sepsis. Lung and abdomen were the most common sites of IFI. *C. albicans*, *C. tropicalis*, and *C. glabrata *were the predominant species and comprised 90% of all isolated strains. Mechanical ventilation (>3 days), APACHE II score, infection with both Gram-positive and Gram-negative bacteria, and urinary catheterization (>3 days) were identified as independent risk factors for IFI in patients with severe sepsis. Compared with the control patients with severe sepsis but without IFI, the matched patients with severe sepsis and IFI had higher hospital mortality, ICU LOS, hospital LOS and hospital costs, and received more aggressive interventions.

We found a high incidence of IFI (28.3%) in critically ill surgical patients suffering from severe sepsis. This is higher than that reported in the SOAP study [4], which identified fungal infection in approximately 17% of the critically ill septic patients in several European countries. This may be because the cohort we studied was more susceptible to fungal infection, as factors that may compromise the integrity of gastrointestinal mucosa and facilitate fungal translocation (such as poor nutrition, trauma, hypotension and therapy with steroids, as well as ischemia and reperfusion) are common in the ICU population. Moreover, injury, trauma, and blood loss in surgical patients, which result in marked depression in cell-mediated immunity, may specifically be associated with high incidence of IFI [26]. However, some factors other than underlying disease and characteristics of patients admitted to the ICU may also contribute to the high incidence. First, in accordance with the criteria used in this study, some of the patients were diagnosed as having IFI but without biopsy, which may lead to enrollment of patients with fungal colonization and overestimation of IFI in surgical patients with severe sepsis. Secondly, most of the enrolled patients with severe sepsis had been hospitalized in the surgical wards before their ICU admission and were routinely administered prophylactic antibiotic treatment; hence, they were more susceptible to nosocomial fungal infection. Third, lung is the most commonly impaired organ in severe sepsis, and mechanical ventilation is instituted in most patients suffering from severe sepsis. Consequently, these individuals are prone to pulmonary fungal infection, because endotracheal tubes may facilitate the intrusion of endogenous or exogenous fungal organisms. In the present study, lung was the major site of fungal infection, and 82.3% of IFI patients who suffered from pulmonary fungal infection received mechanical ventilation for at least 3 days. Finally, the higher occurrence of IFI may also reflect a horizontal transmission of fungal infection in the ICU, because a previous study [27] found *Candida *spp. to be present on the hands of 39% of surgical ICU staff.

Lack of DNA analysis of fungal isolates prevented interpretation, but our findings are in accordance with those of a previous study [28] that identified a 23.96% incidence of fungal infection among patients admitted to an ICU in a Chinese university hospital. Therefore, infection control in ICUs within China may require improvement and standardization. More attention should be given to sanitary precautions in ICU in order to prevent fungal infection; such precautions include using high-efficiency particulate air filters, changing the breathing circuits of ventilators periodically, and applying endotracheal tubes with a dorsal lumen to allow drainage of respiratory secretions. Standard protocols should be established to evaluate the appropriateness of administered aggressive interventions.

The spectrum of fungal species identified in this study was consistent with that in previous studies [29-31], which found that *C. albicans *and non-*albican *species accounted for approximately half of reported cases of IFI. We found four factors to be independently associated with risk for IFI in severe sepsis (mechanical ventilation [>3 days], APACHE II score, infection with both Gram-positive and Gram-negative bacteria, and urinary catheterization [>3 days]). This is congruent with the findings of previous studies focusing on fungal infection in critical illness [32-34], except for coexisting infection with both Gram-positive and Gram-negative bacteria. The latter may be associated with greater severity of infection or use of broad-spectrum antibiotics. However, logistic regression did not identify combination antibiotic therapy as an independent factor influencing the occurrence of IFI in this study. In the present study, combination antibiotic therapy was administered to 50% of the patients with IFI before the onset of IFI, which was similar to the proportion in patients without IFI during their ICU stay (57%, *P *= 0.263).

In order to minimize the influence of confounding factors in our evaluation of the relationship between IFI and outcome, a matched cohort study was conducted. Among the 90 patients with IFI, the 60 patients who could be matched to a control patient were similar to the 30 unmatched patients in terms of age, hospital LOS, ICU LOS, and hospital mortality, which indicates that these matched patients are representative of the whole group of IFI patients. Compared with the control patients with severe sepsis, the matched patients with severe sepsis and IFI had significantly greater hospital mortality. Therefore, this matched cohort study allowed rational estimation that IFI in patients with severe sepsis in surgical ICUs is associated with an excess risk for hospital death of 20%, which is close to that reported in previous correlative studies [35-37]. Patients with IFI also had longer ICU and hospital LOSs than did control patients. Although the present study showed that IFI did not contribute to excess daily costs, it did correlate with excess hospital costs and consumption of medical resources as a result of the prolonged ICU and hospital LOSs.

A major advantage of our study is that a matched cohort study was conducted, and hence the fungus-related mortality was apparent. However, there are several limitations of this study. First, patients diagnosed as having IFI but without biopsy conformation were enrolled, in accordance with the criteria used, which may to a certain degree cause over-diagnosis of IFI. However, there is no consensus definition on IFI in non-neutropenic critically ill patients. Because of hemodynamic and/or respiratory insufficiency and coagulopathy of critical illness, and refusal by most of the Chinese families to allow antemortem or postmortem biopsy, it was rather difficult to diagnose IFI by positive culture from normally sterile sites, especially lower respiratory tract. Exclusion of all patients without biopsy conformation would inevitably have resulted in severe underestimation of IFI in the study population and altered the spectrum of fungal species identified. Therefore, we employed criteria to diagnose IFI patients without biopsy confirmation partly based on the findings of previous studies. Although our criteria may lead to over-diagnosis IFI in the study cohort, the findings based on it may be more objective. Second, this was a retrospective cohort study, although the database was constructed prospectively. Third, selection of control patients was mainly based on severity of illness at admission, as indicated by APACHE II score, but the severity of illness might have drifted apart between case patients and control patients since before the onset of IFI [38]. However, Blot and coworkers [39] found that expected mortality estimated from characteristics obtained on the first day of ICU admission correlated well with observed mortality in candidemic patients. Finally, this study included a relatively small sample to define the characteristics of IFI in a cohort with severe sepsis, in the most populous country in the world. However, there are no national hospital databases of sepsis available on the internet in today's China, and the financial and personnel resources supporting the present study were limited, both of which prevented our team from extending the study to more medical centers on a greater geographic scale.

## Conclusion

The present study found IFI to be frequent in patients with severe sepsis in surgical ICUs, and to be associated with excess risk for death in hospital and greater consumption of medical resources. Therefore, in the process of diagnosing and treating severe sepsis in the ICU, attention should be given to the identification of patients who are at high risk for IFI, as well as prevention of and early intervention in IFI in these patients.

## Key messages

• Ninety severe septic patients (28.3%) were identified as patients with IFI.

• Mechanical ventilation (>3 days), APACHE II score, coexisting infection with both Gram-positive and Gram-negative bacteria, and urethral catheterization (>3 days) were identified as independent risk factors of IFI in patients with severe sepsis.

• IFI is associated with excess risk for death in the hospital and greater consumption of medical resources.

## Abbreviations

APACHE = Acute Physiology and Chronic Health Evaluation; IFI = invasive fungal infection; LOS = length of stay; SOAP = Sepsis Occurrence in Acutely Ill Patients; SOFA = Sequential Organ Failure Assessment.

## Competing interests

The authors declare that they have no competing interests.

## Authors' contributions

G-HX, X-MF, and B-LC contributed to the design of the study and drafted the manuscript. QF, X-MW, Y-HJ, J-LW, Q-LG, M-NG, Q-PX, D-XW, S-LY, S-YY, Z-HD, and Y-BS obtained the data. G-HX, X-MF, B-LC, H-HW, and S-JW participated in data analysis and interpretation of the results.

**Figure 1:**
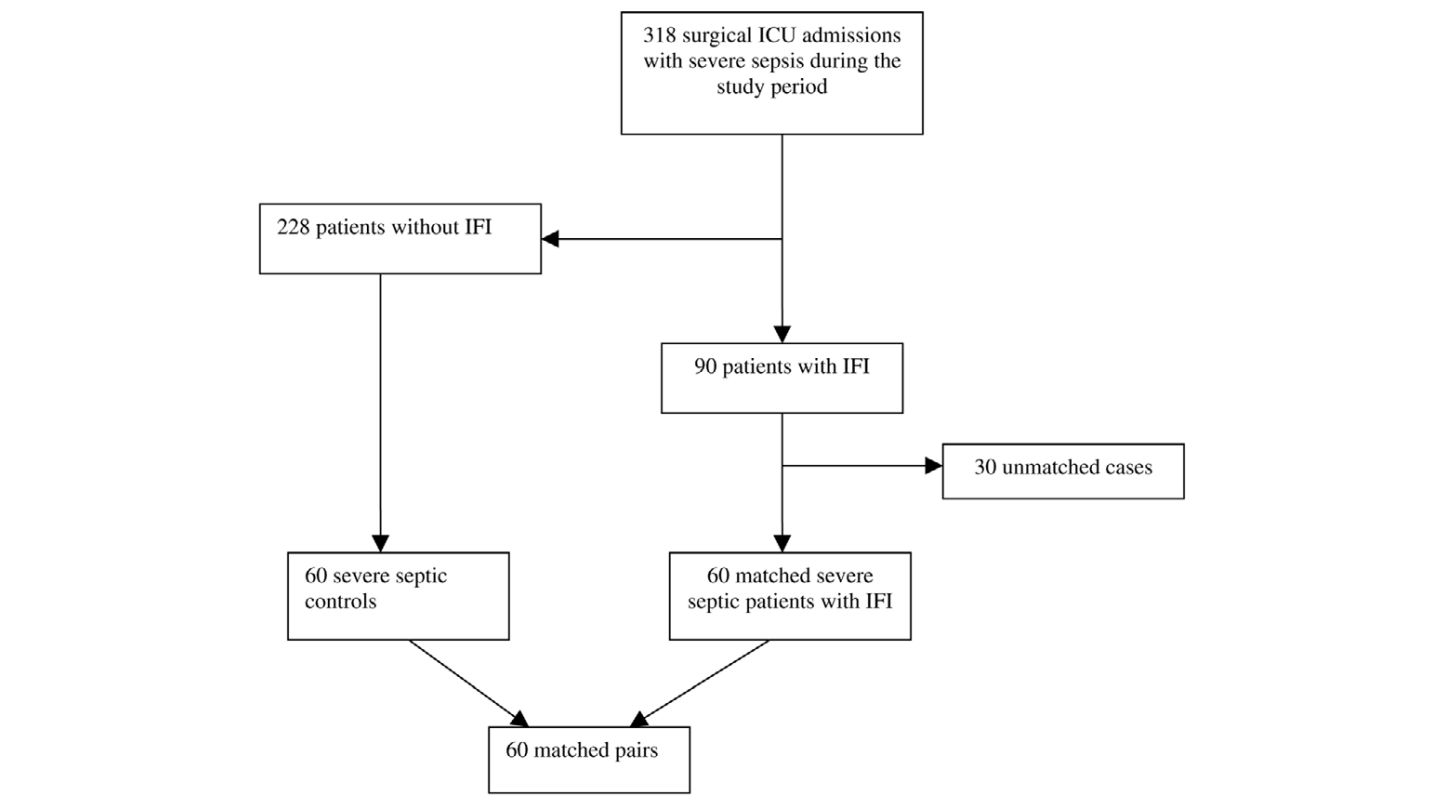
Flow diagram of the enrolled patients. Of the 318 enrolled patients with severe sepsis, 90 were identified as patients with invasive fungal infection (IFI), 60 of whom were 1:1 matched to control patients with severe sepsis but without IFI for unit, sex, age (± 10 years) and Acute Physiology and Chronic Health Evaluation II score (± 3 points). ICU, intensive care unit.
